# Microbial community of predatory bugs of the genus *Macrolophus* (Hemiptera: Miridae)

**DOI:** 10.1186/1471-2180-12-S1-S9

**Published:** 2012-01-18

**Authors:** Thijs Machtelinckx, Thomas Van Leeuwen, Tom Van De Wiele, Nico Boon, Winnok H De Vos, Juan-Antonio Sanchez, Mauro Nannini, Godelieve Gheysen, Patrick De Clercq

**Affiliations:** 1 Laboratory of Agrozoology, Department of Crop Protection, Faculty of Bioscience Engineering, Ghent University, Ghent, Belgium; 2 Laboratory of Microbial Ecology and Technology (LabMET), Faculty of Bioscience Engineering, Ghent University, Gent, Belgium; 3 Laboratory of Bio-imaging and Cytometry, Department of Molecular Biotechnology, Faculty of Bioscience Engineering, Ghent University, Gent, Belgium; 4 Center for Nano- and Biophotonics (NB-Photonics), Ghent University, Gent, Belgium; 5 Instituto Murciano de Investigación y Desarrollo Agrario y Alimentario (IMIDA), Departamento de Biotecnología y Protección de Cultivos, La Alberca, Murcia, Spain; 6 AGRIS Sardegna - DIRVE, Cagliari, Italy; 7 Laboratory of Applied Molecular Genetics, Department of Molecular Biotechnology, Faculty of Bioscience Engineering, Ghent University, Ghent, Belgium

## Abstract

**Background:**

The predatory mirids of the genus *Macrolophus* are key natural enemies of various economically important agricultural pests. Both *M. caliginosus* and *M. pygmaeus* are commercially available for the augmentative biological control of arthropod pests in European greenhouses. The latter species is known to be infected with *Wolbachia* -inducing cytoplasmic incompatibility in its host- but the presence of other endosymbionts has not been demonstrated. In the present study, the microbial diversity was examined in various populations of *M. caliginosus* and *M. pygmaeus* by 16S rRNA sequencing and denaturing gradient gel electrophoresis.

**Results:**

Besides *Wolbachia*, a co-infection of 2 *Rickettsia* species was detected in all *M. pygmaeus* populations. Based on a concatenated alignment of the *16S rRNA* gene, the *gltA* gene and the *coxA* gene, the first is phylogenetically related to *Rickettsia bellii*, whereas the other is closely related to *Rickettsia limoniae*. All *M. caliginosus* populations were infected with the same *Wolbachia* and *limoniae*-like *Rickettsia* strain as *M. pygmaeus*, but did not harbour the *bellii*-like *Rickettsia* strain. Interestingly, individuals with a single infection were not found. A PCR assay on the ovaries of *M. pygmaeus* and *M. caliginosus* indicated that all endosymbionts are vertically transmitted. The presence of *Wolbachia* and *Rickettsia* in oocytes was confirmed by a fluorescence *in situ* hybridisation. A bio-assay comparing an infected and an uninfected *M. pygmaeus* population suggested that the endosymbionts had minor effects on nymphal development of their insect host and did not influence its fecundity.

**Conclusion:**

Two species of the palaearctic mirid genus *Macrolophus* are infected with multiple endosymbionts, including *Wolbachia* and *Rickettsia*. Independent of the origin, all tested populations of both *M. pygmaeus* and *M. caliginosus* were infected with three and two endosymbionts, respectively. There was no indication that infection with endosymbiotic bacteria had a fitness cost in terms of development and fecundity of the predators.

## Background

In recent years, an increasing number of endosymbiotic bacteria have been detected in arthropods, often having intimate associations with their host. In some cases, these bacteria are obligatory for the survival and development of their host, providing them with essential nutrients [1,2], while other endosymbionts are facultative and benefit their hosts’ fitness by protecting them from parasites and diseases [3]. However, some arthropod endosymbionts are considered as ‘reproductive parasites’ [4]. These parasites manipulate the reproduction of their host to promote their own propagation, but these alterations may affect the fitness of their host [5].

The best studied and most widely spread arthropod endosymbiont is *Wolbachia*, an obligate intracellular Alpha-proteobacterium that infects approximately 66% of all insects [6]. *Wolbachia* alters its host in various ways, of which cytoplasmic incompatibility (CI) is probably most studied [7]. Cytoplasmic incompatibility occurs when an uninfected female mates with an infected male (unidirectional CI) or when an infected female mates with an infected male bearing another *Wolbachia*-strain (bidirectional CI). This cross results in embryonic death, while all other crosses produce normal progeny. Other manipulations of *Wolbachia* are male killing, in which infected male embryos die [8], parthenogenesis, in which nonfertilized infected mothers only produce infected female offspring [9] and feminization, in which genetic males are converted into fertile females [10]. In rare cases, *Wolbachia* is obligate for its insect host: in the parasitoid wasp *Asobara tabida*, the bacterium is necessary for oogenesis completion [11].

Besides *Wolbachia*, a wide range of other inherited bacteria are currently being investigated. One of these symbionts, C*ardinium*, [12] does not infect as many arthropods as *Wolbachia*, but can affect its host almost as strikingly by causing CI, parthenogenesis and feminization [13-15]. Other important endosymbionts manipulating the reproduction of their host include *Spiroplasma*, *Arsenophonus*, *Flavobacterium* and *Rickettsia*.

Insights into the importance of *Rickettsia* as a reproductive parasite are increasing rapidly [16]. *Rickettsia* bacteria are Alpha-proteobacteria closely related to *Wolbachia* and are best known as arthropod-borne vertebrate pathogens. One *Rickettsia* is a known plant pathogen, causing papaya bunchy top disease vectored by a leafhopper [17]. In recent years, however, an increasing amount of non-pathogenic ‘arthropod *Rickettsia*’ have been discovered, lacking a secondary host. These endosymbionts are dispersed throughout different arthropod classes, including a wide range of insect species [18]. Although their biological role needs to be largely elucidated, these ‘arthropod *Rickettsia*’ can act as reproductive parasites. In the ladybirds *Adalia bipunctata* and *Adalia decempunctata* as well as in the buprestid beetle *Brachys tessellatus* the endosymbiont has been demonstrated to cause male embryonic lethality [19-21]. Further, parthenogenesis induction is described in the parasitoid wasps *Pnigalio soemius* and *Neochrysocharis formosa *[22,23]*.* Perotti *et al. *[24] also found evidence of an obligate *Rickettsia* in the booklouse *Liposcelis bostrychophila* with a key role for egg production.

Endosymbiotic bacteria have been described in harmful as well as beneficial arthropods. The presence and role of endosymbionts are well studied in certain groups of beneficial arthropods, including hymenopteran parasitoids and coccinellid predators [25]. However, relatively few studies have focused on the endosymbiotic bacteria of predatory Heteroptera (true bugs), despite their economic importance as biological control agents of agricultural pests [26]. In the present study, the microbial community of *Macrolophus* spp. is examined. *Macrolophus* is a genus of polyphagous mirid predators commonly used in European greenhouses for the biological control of whiteflies, spider mites, thrips, aphids, and leaf miners [27,28]. The two major species that have been used in commercial biological control are *M. caliginosus* and *M. pygmaeus*. It has been established that *M. pygmaeus* carries *Wolbachia*, which induces strong CI in its host and may thus have a substantial impact on the practical use of the predator in programmes of biological pest control [29]. However, other endosymbiotic bacteria have not been demonstrated to infect *Macrolophus* spp. The microbial population of *M. pygmaeus* and *M. caliginosus* was examined by *16S rRNA* gene sequencing and denaturing gradient gel electrophoresis (PCR-DGGE). The latter technique has been used to characterize complex bacterial compositions of environmental samples [30,31], but also proved useful to explore bacterial communities in arthropods [32-34]. Furthermore, a fluorescence *in situ* hybridization (FISH) analysis was performed to visualize the co-localization of different endosymbionts. Improving our understanding of the composition and functions of the endosymbiotic community of these predatory insects may contribute to optimizing their use as natural enemies of agricultural pests.

## Methods

### Insect populations

Adults of different *Macrolophus* populations were collected from sites in Greece, Spain and Italy (Table 1) and preserved in 70% ethanol. A laboratory strain of *M. pygmaeus* originating from Koppert B.V. (Berkel en Rodenrijs, The Netherlands) and an endosymbiont-free strain (cured by tetracycline) which originated from the same stock culture [29] was maintained in the Laboratory of Agrozoology of Ghent University since 2006 and 2008, respectively. The endosymbiont-free strain was cured by feeding it on an artificial diet containing tetracycline for 13 generations [29]. From the next generation on, this population was supplied with frozen eggs of the Mediterranean flour moth *Ephestia kuehniella* (also from Koppert B.V). A PCR-assay using endosymbiont-specific primers (Table 2) was performed (every 3 to 4 generations) to ensure its cured status. A laboratory population of *M. caliginosus* was established based on field collected individuals in Santa Margherita di Pula, Sardinia, Italy.

Both *Macrolophus* spp. were reared in Plexiglas cylinders (9 cm diameter, 3.5 cm high) at 23°C, 65% relative humidity and a 16 : 8 light : dark (L : D) h photoperiod. A small bell pepper plant (*Capsicum annuum* L. cv. California Wonder) was used as an oviposition substrate and a source of moisture [28]. The predator was fed with frozen *E. kuehniella* eggs which were replenished every 2 days.

**Table 1:** *Macrolophus* spp. populations used in this study.

Strain name	Origin	Host plant	Species	Accession no.
AmaDV	Amaliada, Greece	*Dittrichia viscosa*	*M. caliginosus*	HE583190
AmaSN	Amaliada, Greece	*Solanum nigrum*	*M. pygmaeus*	HE583191
Esp	La Vereda, Murcia, Spain	*Solanum lycopersicum*	*M. pygmaeus*	HE583192
Grec	Thessaloniki, Greece	*S. nigrum*	*M. pygmaeus*	HE583193
KorDV	Korinthos, Greece	*D. viscosa*	*M. caliginosus*	HE583194
KorSN	Korinthos, Greece	*S. nigrum*	*M. pygmaeus*	HE583195
Kp	Laboratory strain, originating from Koppert BV	*Capsicum annuum*	*M. pygmaeus*	HE583196
KypDV	Kyparissia, Greece	*D. viscosa*	*M. caliginosus*	HE583197
KypSN	Kyparissia, Greece	*S. nigrum*	*M. pygmaeus*	HE583198
Sard	Santa Margherita di Pula, Sardinia, Italy	*D. viscosa*	*M. caliginosus*	HE583199
Skyd	Skydra, Greece	*S. nigrum*	*M. pygmaeus*	HE583200
ThivDV	Thiva, Greece	*D. viscosa*	*M. pygmaeus*	HE583201

### DNA extraction

Male and female adults were surface sterilized in 70% ethanol and rinsed with sterilized water. Individuals from laboratory-reared populations were starved for 24h before extraction to allow voiding of the gut content. A DNeasy Blood and Tissue Kit (Qiagen, Venlo, The Netherlands) was used to extract the DNA, applying the manufacturer’s instructions for gram-positive bacteria. A no-template control and DNA from the cured strain was also included in each DNA-extraction to prevent false positive results in the PCR and PCR-DGGE reactions. DNA was eluted in 50 µl of DNeasy buffer AE (10 mM Tris-Cl, 0.5 mM EDTA, pH 9.0) after which DNA-quality was checked by staining a 1% agarose gel in 0.5 x TAE with ethidium bromide and visualizing with UV-illumination (Bio-Rad Gel Doc XR System, 254 nm; Bio-Rad, Hercules, CA, USA). DNA-concentration was measured with the Nanodrop ND-1000 spectrophotometer (Thermo Fisher Scientific, Wilmington, DE, USA).

Ovaries and guts were dissected in a vertical laminar flow and washed twice with sterilized water under a stereomicroscope. Between 20 to 30 guts or ovaries were used for the DNA-extraction.

### Primers

All primers used in this study are listed in Table 2. *Macrolophus s*pecies determination was clarified by targeting a part of the cytochrome b gene [35]. The bacterial community was characterized in *M. pygmaeus* by using universal primers 27F-806R and 27F-1525R which amplify the bacterial *16S rRNA* gene. Specific *Rickettsia*-primers targeting the *16S rRNA* gene were constructed using primer3 [36] as implemented in primer-BLAST [http://www.ncbi.nlm.nih.gov/]. The primer pair Rick1F-1492R amplified a part of both *Rickettsia* species, whereas the *Wolbachia* primers were based on the *wsp* gene (Table 2).

**Table 2:** Primer sequences used in this study for PCR and PCR-DGGE. The accession numbers point to the genes that were used to construct the gene specific primers.

Targeted gene	Name	Sequence	Accession number/ Reference
Cytochrome b gene of *Macrolophus* spp.	CB-1	5’- TATGTACTACCATGAGGACAAATATC -3’	[68]
	CB-2	5’- ATTACACCTCCTAATTTATTAGGAAT -3’	[68]
	Lau1F	5’- AATGGCTATGAGGGGGRTTCTC -3’	[35]
General primers for the bacterial *16S rRNA* gene	27F	5’- AGAGTTTGATCMTGGCTCAG -3’	[43]
	806R	5’- GGACTACCAGGGTATCTAAT -3’	[69]
	1492R	5’- TACGGYTACCTTGTTACGACTT -3’	[43]
	1525R	5’- AAAGGAGGTGWTCCARC -3’	[69]
V3 region of the bacterial *16S rRNA* gene*	338F^GC^	5’- **CGCCCGCCGCGCGCGGC**	[43]
		**GGGGCGGGGGCACGGGGGG**	
		ACTCCTACGGGAGGCAGCAG -3’	
	518R	5’- ATTACCGCGGCTGCTGG -3’	[30]
*wsp* gene of *Wolbachia*	wsp81F	5’- TGGTCCAATAAGTGATGAAGAAAC -3'	[70]
	wsp691R	5’- AAAAATTAAACGCTACTCCA -3’	[70]
*16S rRNA* gene of *R. limoniae* and *R. bellii*	Rick-1F	5’- ATACCGAGTGRGTGAYGAAG -3’	AF322442, L36103
*16S rRNA* gene of *R. limoniae*	Ricklimoniae-F	5’- CGGTACCTGACCAAGAAAGC -3’	AF322442
*16S rRNA* gene of *R. bellii*	Rickbellii-R	5’- TCCACGTCGCCGTCTTGC -3’	L36103
Citrate synthase gene (gltA)	gltA133f	5’- GGTTTTATGTCTACTGCTTCKTG -3’	[17]
	gltA1197r	5’- CATTTCTTTCCATTGTGCCATC- 3’	[17]
Cytochrome c oxidase gene (coxA)	coxA322f	5’- GGTGCTCCTGATATGGCATT -3’	[18]
	coxA1413r	5’- CATATTCCAACCGGCAAAAG -3’	[18]
p-GEMT cloning vector	T7	5’- TAATACGACTCACTATAGGG -3’	Promega
	SP6	5’- CTATTTAGGTGACACTATAG -3’	Promega

*The sequence of the GC-clamp is indicated in bold

### PCR and cloning

All PCR reactions were executed using a Biometra TProfessional Standard Gradient Thermocycler (Westburg, Leusden, The Netherlands) in 50 µl containing 2 mM MgCl, 0.2 mM deoxynucleotide triphosphate (dNTP) mix (Invitrogen, Carlsbad, CA, USA), 2 mM MgCl_2_, 5 µl 10x PCR-buffer (Invitrogen), 1 U Taq DNA polymerase (Invitrogen) and 1 µl DNA template (between 100 and 200 ng/µl). PCR for species determination was executed under the following conditions [35]: 5 min at 95 °C, 36 cycles of 45 s at 95 °C, 30 s at 50 °C, 30 s at 72 °C and a final extension of 10 min at 72 °C. Amplification conditions for all other PCR reactions were 2 min at 94 °C, 35 cycles of 30 s at 94 °C, 45 s at 54 °C, 1 min 30 s at 72 °C and a final elongation step of 5 min at 72 °C. PCR products were electrophoresed on a 1 % agarose gel in 0.5 x TAE-buffer and after staining with ethidium bromide visualized under UV-light (Bio-Rad Gel Doc XR System, 254 nm). PCR products were purified using the EZNA Cycle Pure Kit (Omega Bio-Tek Inc., Norcross, GA, USA). If necessary, purified PCR products were cloned into the pGEM-T Vector (Promega, Madison, WI, USA) and transformed in *Escherichia coli* DH5α cells. Plasmids containing inserts with expected sizes were selected and sequenced with SP6/T7 primers (Table 2) by LGC Genomics (Berlin, Germany). Sequences were submitted to the EMBL Nucleotide Sequence Database.

### Phylogenetic analysis of the *Rickettsia* endosymbionts

DNA sequences of the amplified *Rickettsia* species were aligned with *Rickettsia* sequences found via BLASTN-searches against the NCBI nucleotide (nr) databank [37]. Alignments were made with ClustalW as implemented in BioEdit [38]. A concatenated alignment of three genes was constructed, using the *16S rRNA* gene, the citrate synthase gene (gltA) and the cytochrome c oxidase I gene (coxA). Genes used for constructing the phylogenetic tree are summarized in additional file 1. Missing data was allowed in our alignment, as not all three genes have been sequenced for all used *Rickettsia* sequences [18]. Phylogenetic reconstruction was performed under Bayesian Maximum Likelihood Inference, using Mr. Bayes version 3.1.2 [39]. The model of evolution was chosen with MrModeltest version 2.2 [40] and the Akaike information criterion. The general time reversible (GTR) + invariant sites (I) + gamma distribution (G) model was chosen, in which 10^6^ generations were analyzed, sampling trees every 100 generations. The first 2500 trees were discarded as ‘burn-in’. *Orientia tsutsugamushi* was chosen as the outgroup. All trees were visualized in Treeview [41].

### Denaturing Gradient Gel Electrophoresis (PCR-DGGE)

A PCR-DGGE was performed using the hypervariable V3-region of the *16S rRNA* gene. For this purpose, genomic DNA was extracted from male and female adults from the collected *M. pygmaeus* and *M. caliginosus* populations and from a tetracycline-cured strain of *M. pygmaeus*. Five to ten adults were pooled for each population. First, a PCR-DGGE was carried out using a non-nested PCR approach with primer pair 318F-518R (Table 2) in 50µl reaction mixtures as described above. Amplification conditions were: 95 °C for 5 min, followed by 33 cycles of 95 °C for 30 s, 55 °C for 45 s, 72 °C for 1 min 30 s and a final elongation of 65 min at 72 °C to avoid artifactual double bands [42]. However, this approach also amplified the *18S rRNA* gene of *Macrolophus* spp. (data not shown). The high amplification of this gene can suppress the detection of bacteria with a low titer. Consequently, a semi-nested PCR was carried out on all populations to avoid the *Macrolophus* 18S rDNA band showing up in the PCR-DGGE-profile. The semi-nested PCR was carried out using the 27F-primer, which is widely used for the molecular detection of bacteria [43,44]. The first PCR-step was performed using the primer pair 27F-518R with amplification conditions: 5 min at 95 °C, 13 cycles of 30 s at 95 °C, 45 s at 55 °C, 1 min 30 s at 72 °C and a final elongation of 65 min at 72 °C. Next, 1 µl of each product was used in a touchdown PCR reaction with primers 338f-518R with a profile of 5 min at 95 °C, 10 cycles of 30 s at 95 °C, 45 s at (60 °C – 0.5 °C), 1 min 30 s at 72 °C, 13 cycles of 30 s at 95 °C, 45 s at 55 °C, 1 min 30 s at 72 °C and a final elongation step of 65 min at 72 °C. This PCR-DGGE provided a similar profile as the non-nested PCR-DGGE, but the eukaryotic *18S rRNA* gene was absent. The empty lane of the no-template control indicated the absence of contamination. The Bio-Rad DCode system was used for the analysis. Gels with 8 % (w/v) polyacrylamide were ran in 1 x TAE (40 mM Tris-Cl, 20 mM glacial acetic acid, 1 mM disodium EDTA.2H_2_O, pH 7.4) with a denaturing gradient of 45 to 60 % (100 % denaturant contains 7 M urea and 40% formamide) for 16 h at 38 V. Gels were stained with SYBR-Green and visualized under UV light (Isogen ProXima 16 Phi system, Isogen Life Science, Sint-Pieters-Leeuw, Belgium). To analyze the different bands of the DGGE-pattern, bands were excised from gel, and washed for three times in sterile water. DNA was then eluted from the gel by heating at 37 °C with 100 µl of sterile water; 1 µl was used for reamplification. PCR-products were cloned in the pGEM-T vector, reamplified using primer pair 338F-518R and run on a PCR-DGGE gel to discriminate the different bands. Plasmids corresponding to bands of interest were sent to LGC genomics for sequencing.

### Fluorescence *in situ* hybridisation

The co-localization of *Rickettsia* and *Wolbachia* in the reproductive tissues was confirmed with a fluorescent *in situ* hybridization (FISH). The analysis was carried out following the protocol of Crotti et al. [45] for whole-mounted samples with slight modifications. Ovaries of infected and cured *M. pygmaeus* females were collected in a drop of 1 x PBS under a stereomicroscope, fixed for 1 h in 4 % paraformaldehyde in 1 x PBS and washed three times with 1 x PBS. The ovaries were then incubated for 1 min in a 100 µg/ml pepsin solution and washed again three times with 1 x PBS and one time with the hybridization buffer without probe (2 x SSC, 50 % formamide). Hybridization was carried out overnight at 46°C in hybridization buffer with 10 pmol/ml fluorescent probe. The next day, samples were washed in hybridization buffer without probe, two times in 0.1 x SSC and two times in 1 x PBS. Subsequently, the samples were whole-mounted with Vectashield Mounting Medium (Vector Labs, Burlingame, CA, USA) and images were acquired using a Nikon A1R confocal microscope, mounted on a Nikon Ti body, using a 60 x (NA1.4) oil objective. Probes used for the analysis were the *Rickettsia*-specific probe Rb1-Cy3 [33] (5’-Cy3-TCCACGTCGCCGTCTTGC-3’), and both WOL2 [46] (5’-Cy5-CTTCTGTGAGTACCGTCATTATC-3’) and WOL3 [47] (5’-Cy5-AACCGACCCTATCCCTTCGAATA-3’), targeting *Wolbachia*. A no-probe experiment and the hybridization of an aposymbiotic ovariole was executed as a specifity control.

### Fitness effects

To investigate the effect of the endosymbionts on the fitness of *M. pygmaeus*, nymphal development and fecundity of the predator were compared between the infected laboratory-strain of *M. pygmaeus* and an endosymbiont-free *M. pygmaeus* population. The general procedure largely follows the method of Vandekerkhove et al. [48], with slight modifications. First instars (<24h) of the 39^th^ generation of each population were individually caged in vented plastic cups (4 cm diameter and 2.5 cm high) containing a wax paper drenched in paraffin. A parafilm dome filled with water and *E. kuehniella* eggs were provided as a source of water and food, respectively. Water domes and eggs were replaced every two days. Nymphs which died on the first or second day of the experiment were replaced by new ones, assuming that their death was caused by handling. Nymphal development and survival were checked daily. Nymphs that successfully reached the adult stage were sexed and weighed at emergence (i.e., within 24 h after moulting). Adult pairs were then transferred to a new plastic cup containing a tobacco leaf disc placed with the upper side on a 1 % agar layer. Two crosses were tested: infected males with infected females [I♂ x I♀] and uninfected males with uninfected females [U♂ x U♀]. Eggs of *E. kuehniella* were offered as a food source for the adult predators, whereas the tobacco leaf served as a source of moisture and an oviposition substrate. After 7 days, females were dissected and oocytes were counted [28]: late vitellogenic to mature oocytes were scored 1; early to mid vitellogenic oocytes 0.5 and previtellogenic oocytes 0.25. Mature oocytes present in the oviducts were also scored as 1. The scores for all ovarioles were then summed providing a weighted sum of oocytes, which can reliably be used to predict the lifetime fecundity of *M. pygmaeus *[28]. Furthermore, the leaf discs were immersed in safranin and screened for oviposited eggs. Effects of infection status on nymphal development, adult weight and fecundity were statistically examined by a one-way analysis of variance (ANOVA) or a Mann-Whitney U Test using SPSS 17.0 [49].

## Results

### Insect species collection and identification

The *Macrolophus* populations from Greece and Italy were collected on the wild plants *Solanum nigrum* and *Dittrichia viscosa* which are considered to be conservation host plants for *M. pygmaeus* and *M. caliginosus*, respectively [50,51]*.* Some *M. pygmaeus* populations were also collected on *D. viscosa*, although their survival is reported to be poor on this plant [50]. In Spain, *M. pygmaeus* was also collected on tomato, *Solanum lycopersicum.*

The primer pairs CB1-CB2 and LAU1f-CB2, which both amplify a part of the cytochrome b gene, were used to elucidate the species identity of the collected insects. In accordance with Martinez-Cascales et al. [35], the primer pair CB1-CB2 yielded poor results for *M. caliginosus* DNA; therefore the LAU1F-CB2 primer pair was used for species identification. The latter amplified all *Macrolophus*-DNA, although the LAU1-primer was designed to specifically amplify *M. caliginosus*-DNA [35]*.* Results are summarized in Table 1.

### *16S rRNA* gene sequencing

A PCR assay was carried out on a pool of adult *M. pygmaeus* males and females of the laboratory strain using general primers targeting the bacterial *16S rRNA* gene. A total of 23 clones were sequenced, varying in length depending on the use of primer pair 27F-806R or 27F-1525R (Table 2). These sequences were compared with the non-redundant (nr) nucleotide database at the National Center for Biotechnology (NCBI) using BLASTN. Three of the cloned bacteria can be considered as endosymbionts, namely *Wolbachia* and two *Rickettsia* species (Table 3). The two *Rickettsia* species were identified using the primer pair 27F-806R. In order to obtain approximately 1500 base pairs of their *16S rRNA* gene, a PCR using a forward primer based on the partially known sequences of the two *Rickettsia* species was designed and combined with the general bacterial 1492R primer (Rick1F-1492R, Table 2). One of these *Rickettsia* species exhibited a 99% similarity to *Rickettsia limoniae* and the *Rickettsia* endosymbiont of the water beetle *Deronectes platynotus*. The second one was 99% similar to *Rickettsia bellii* and the *Rickettsia* endosymbiont of the pea aphid *Acyrthosiphon pisum*. Other cloned bacteria are not regarded as endosymbiotic bacteria, but rather as environmental or gut bacteria (Table 3).

**Table 3:** Partial 16S rDNA sequences isolated in this study by cloning and PCR-DGGE. The accession number of the closest relative is indicated between brackets.

Closest known relative	Phylogenetically related class	Sequenced length (bp)	Identity (%)	Accession no.
**16S rRNA PCR cloning of *****M. pygmaeus***				
*Rickettsia limoniae* strain Brugge (AF322443)	Alpha-proteobacteria	1422	99	HE583202
*Rickettsia bellii* (L36103)	Alpha-proteobacteria	1422	99	HE583203
*Wolbachia* endosymbiont of *Culex quinquefasciatus* (AM999887)	Alpha-proteobacteria	1461	98	HE583204
Uncultured bacterium (GQ360069)	Gamma-proteobacteria	1496	99	HE583205
Uncultured bacterium (HM812162)	Firmicutes	767	100	HE583206
Uncultured bacterium (FJ512272)	Firmicutes	764	99	HE583207
Uncultured bacterium (GU118480)	Beta-proteobacteria	743	99	HE583208
PCR-DGGE*				
1) *Wolbachia* endosymbiont of *Polydrusus pilifer* (JF304463)	Alpha-proteobacteria	135	100	HE583209
2) *Rickettsia bellii* (L36103)	Alpha-proteobacteria	135	99	HE583210
3) Uncultured bacterium (JF011887)	Gamma-proteobacteria	160	100	HE583211
4) Uncultured bacterium (JF011887)	Gamma-proteobacteria	160	99	HE583212
5) *Rickettsia limoniae* strain Brugge (AF322443)	Alpha-proteobacteria	137	100	HE583213
6) Uncultured *Streptococcus* sp. (GU132113)	Firmicutes	161	100	HE583214
7) Uncultured bacterium (FN421660)	Gamma-proteobacteria	157	99	HE583215
8) *Rickettsia bellii* (L36103)	Alpha-proteobacteria	135	99	HE583216
9) Uncultured bacterium (JF206698)	Gamma-proteobacteria	160	100	HE583217
10) *Serratia* sp. (HQ891979)	Gamma-proteobacteria	160	100	HE583218
11) *Enterobacter cloacae* (HQ888762)	Gamma-proteobacteria	160	100	HE583219
12) *Serratia* sp. (HQ888762)	Gamma-proteobacteria	160	100	HE583220

*the numbers correspond to the bands in Fig. 2 and Fig. 3

**Figure 1:**
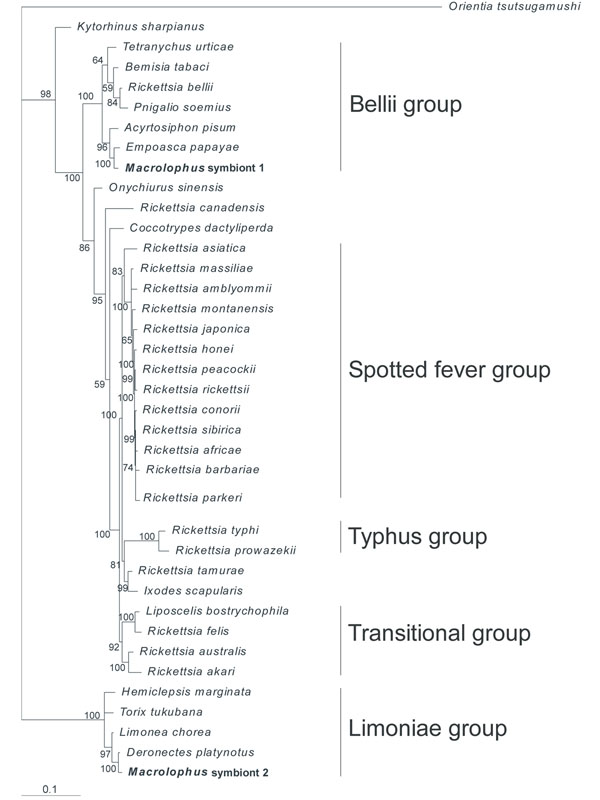
**Phylogenetic tree of *Rickettsia.*** Rooted phylogenetic tree estimated using Bayesian inference of phylogeny, based on concatenated sequences of *16S*, *gltA* and *coxA* of *Rickettsia*. Posterior probabilities supporting nodes (> 50) are shown. The different *Rickettsia-*strains are indicated either as their species name or as their host species. Group names are indicated on the right.

**Figure 2:**
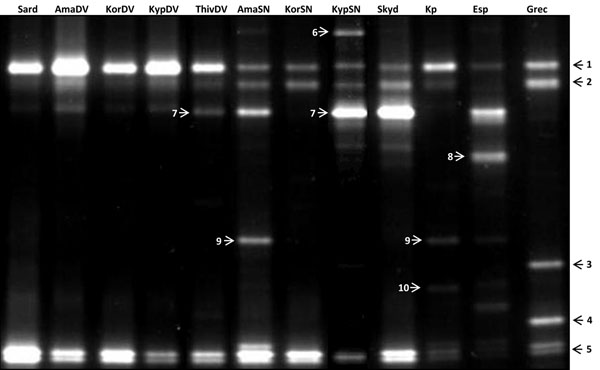
**PCR-DGGE profiles of hypervariable 16 rRNA V3-regions of various *M. pygmaeus* and *M. caliginosus* populations.** Numbers correspond to PCR-DGGE amplicons that were excised from the gel, cloned and sequenced (Table 3).

**Figure 3:**
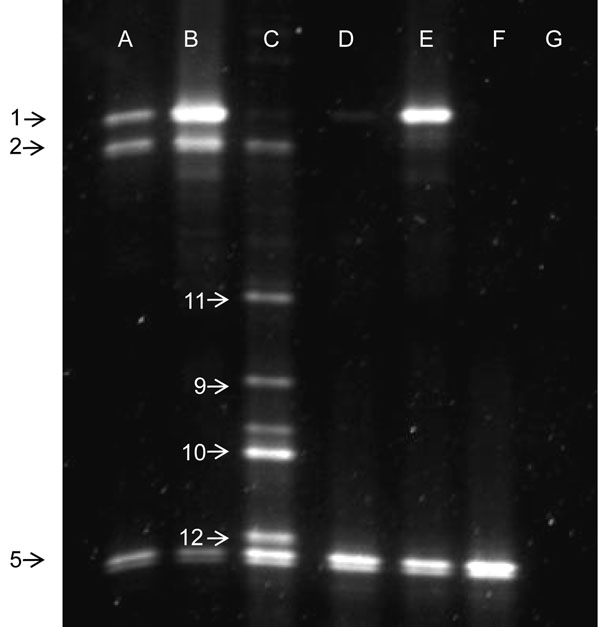
**PCR-DGGE on tissues of *M. pygmaeus* and *M. caliginosus.*** PCR-DGGE profiles of hypervariable 16 rRNA V3-regions of adults, ovaries and guts of the laboratory strains of *M. pygmaeus* and *M. caliginosus*. A: *M. pygmaeus* adults, B: *M. pygmaeus* ovaries, C: *M. pygmaeus* guts, D: *M. caliginosus* adults, E: *M. caliginosus* ovaries, F: *M. caliginosus* guts, G: cured *M. pygmaeus* adults. Numbers correspond to PCR-DGGE amplicons that were excised from the gel, cloned and sequenced (Table 3).

To investigate the presence of similar endosymbionts in the other (wild) populations of *M. pygmaeus* and the closely related species *M. caliginosus*, a PCR assay was performed using *Rickettsia-* (RicklimF-1492R and 27F-RickBelR) and *Wolbachia-*specific primers (Table 2). This assay revealed the presence of all three endosymbionts in all *M. pygmaeus* populations. In addition, *Wolbachia* and a *Rickettsia*-species that was 100% similar to the *R. limoniae-*species of *M. pygmaeus* were detected in all *M. caliginosus* populations. However, the *bellii*-like *Rickettsia* present in *M. pygmaeus* was not found in *M. caliginosus*.

A diagnostic PCR using *Rickettsia*-specific primers and *wsp*-primers on 20 adult males and 20 adult females of the laboratory strain of *M. pygmaeus* showed that all tested individuals were infected with the three endosymbionts. The same experiment was repeated using a *M. caliginosus* strain found on *D. viscosa* in Sardinia, Italy, revealing that all adults were infected with *Wolbachia* and *R. limoniae*. The presence of *Wolbachia* and *Rickettsia* in the ovaries of *M. pygmaeus* and *M. caliginosus* was confirmed by PCR using 20 ovaries of both species.

### Phylogenetic analysis

A Bayesian inference (BI) phylogenetic tree based on a concatenated alignment of the *16S rRNA*, *gltA* and *coxA* genes was constructed to check the phylogeny of the two *Rickettsia* species (Fig. 1). However, the gltA-primers did not amplify the citrate synthase gene of ‘*Macrolophus* symbiont 2’ (Fig. 1).The phylogenetic relationships of the *Wolbachia* strain in *M. pygmaeus* were previously elucidated [29]. The two *Rickettsia* species are related to two different clades. The phylogenetic tree indicated that the first *M. pygmaeus Rickettsia* endosymbiont is associated with the ‘Bellii’ group, clustering with the *Rickettsia* endosymbionts of the two-spotted spider mite *Tetranychus urticae*, the pea aphid *A. pisum* and the tobacco whitefly *Bemisia tabaci*, among others. The second *Rickettsia* endosymbiont is situated in the ancestral ‘Limoniae’ group, clustering with the *Rickettsia* endosymbiont of the water beetle *Deronectes platynotus* and the cranefly *Limonia chorea*.

### Denaturing Gradient Gel Electrophoresis (PCR-DGGE)

PCR-DGGE-profiling targeting the hypervariable V3-region of the *16S rRNA* gene (Table 2) was applied to analyze the microbial community of the studied *M. pygmaeus* and *M. caliginosus* populations. These populations exhibited similar profiles (Fig. 2), as both species had bands with high and low intensity.

These bands were excised from gel, eluted and cloned. After sequencing, BLASTN searches were performed against the nr-database of NCBI. Table 3 summarizes the BLAST-results of the sequenced bands. In corroboration of the cloning experiments using the *16S rRNA* gene, bands with a high similarity to *Wolbachia*, *R. bellii* and *R. limoniae* were found in the *M. pygmaeus* populations, while the PCR-DGGE-profile of *M. caliginosus* lacked the band attributed to the *bellii*-like *Rickettsia*. The other excised bands corresponded to bacteria from the Gamma-proteobacteria and Firmicutes. These bacteria are generally considered as environmental bacteria or micro-organisms related to the digestive tract [23], but their function is unknown in *Macrolophus* spp. The profile of the cured strain only showed the *18S rRNA* band in the non-nested DGGE-PCR (data not shown), and no bands in the nested DGGE-PCR (Fig. 3). One band, corresponding to an uncultured Gamma-proteobacterium, was found in five *Macrolophus* populations.

Furthermore, a PCR-DGGE-profile of the ovaries and the gut of the laboratory strain of *M. pygmaeus* and *M. caliginosus* was generated (Fig. 3). DNA was extracted from a pool of 20-30 dissected ovaries and 20-30 dissected guts, respectively. The PCR-DGGE-profile of the ovaries of *M. pygmaeus* and *M. caliginosus* only showed the bands related to *Wolbachia* and the *Rickettsia* species. The DGGE-profile of the guts showed the presence of the two *Rickettsia* species and the Gamma-proteobacteria, but the band corresponding to *Wolbachia* was very faint.

### FISH

**Figure 4:**
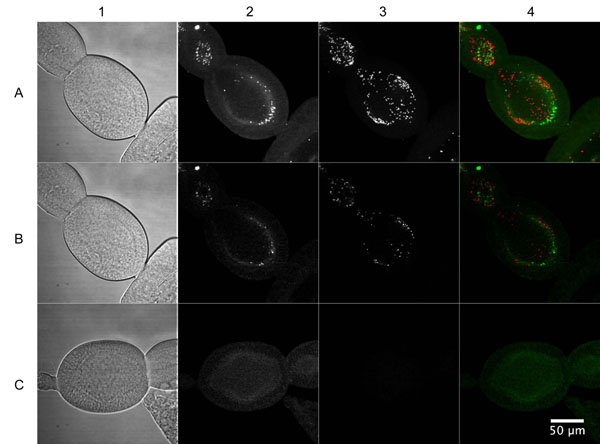
**FISH of infected and uninfected *M. pygmaeus* ovarioles (60 x objective).** All images were acquired using identical settings and the contrast has been adapted equally. A: Maximum intensity projection of 20 confocal sections of an infected *M. pygmaeus* ovariole, B: Optical section of an infected *M. pygmaeus* ovariole, C: Optical section of a cured *M. pygmaeus* ovariole. 1: Bright field channel, 2: *Rickettsia* Cy3 channel, 3: *Wolbachia* Cy5 channel, 4: overlay of *Rickettsia* and *Wolbachia* channel. Green: *Rickettsia*, Red: *Wolbachia.*

**Figure 5:**
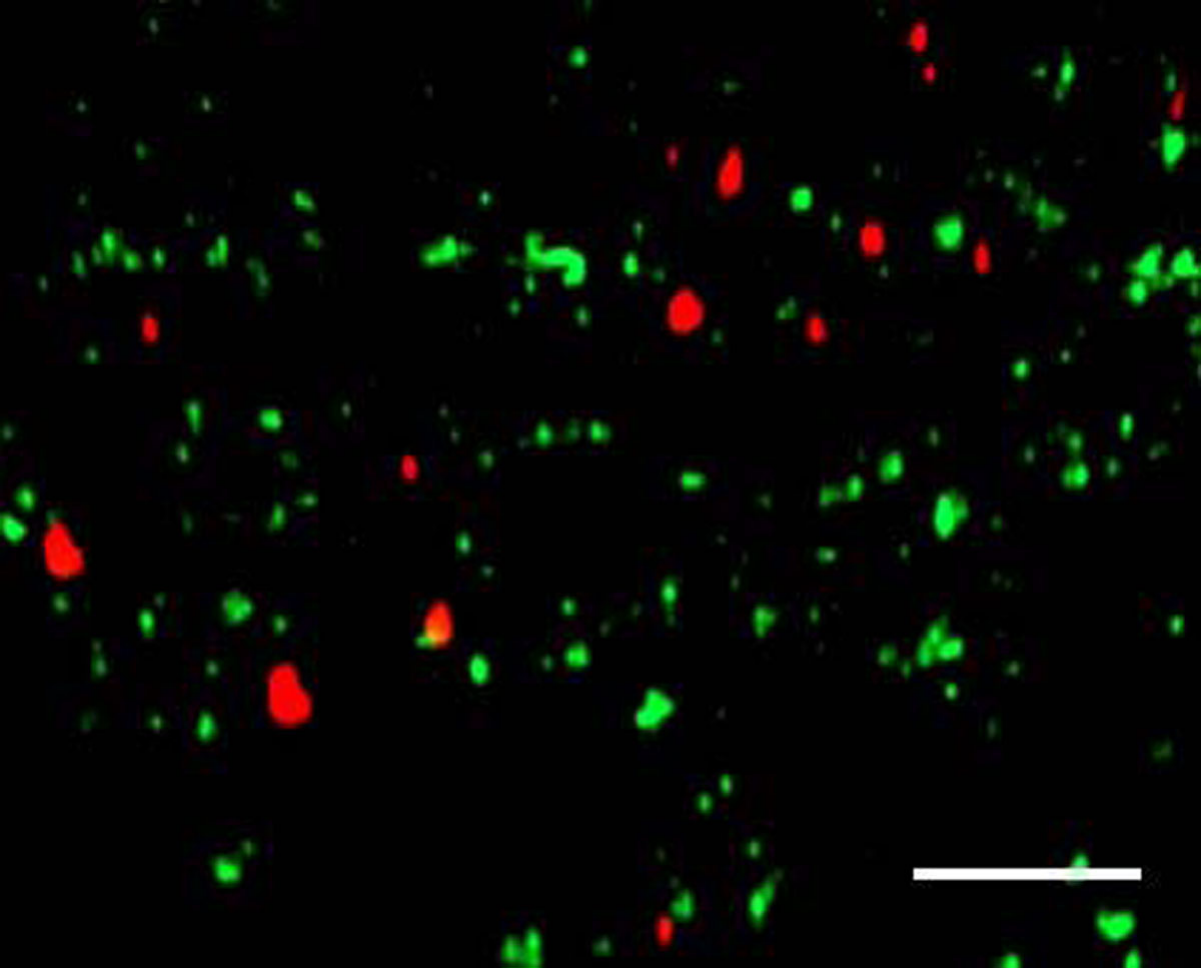
**Volume rendered view of an infected ovariole, showing the colocalization of *Rickettsia* (green) and *Wolbachia* (red).** The picture was made in NIS-viewer (Nikon Instruments Inc., Badhoevedorp, The Netherlands) based on 21 confocal slices. Scale bar = 10µm.

Vertical transmission of the *Wolbachia* and *Rickettsia* endosymbionts was confirmed by FISH analysis on the ovaries of the laboratory strain of *M. pygmaeus*. A high concentration of both *Wolbachia* and *Rickettsia* was observed inside the ovarioles (Fig. 4 A-B), while no infection was detected in a cured ovariole (Fig. 4 C). A 3D-view of a 10 µm cross-section made by a stack of 21 confocal slices provided a clear distinction of single *Rickettsia* and *Wolbachia* bacteria inside the ovarioles (Fig. 5). A no-probe control verified the specific fluorescence of the endosymbionts, as no fluorescence was observed.

### Fitness effects

Bio-assays were carried out to examine potential fitness effects of the endosymbionts on their *Macrolophus* host. In a first experiment, nymphal development was compared between infected and uninfected individuals of *M. pygmaeus*, revealing positive effects of the infection on some developmental traits (Table 4). Infected *M. pygmaeus* males developed significantly faster than cured males (P<0.001). Moreover, infected females were significantly heavier at emergence than uninfected ones (P=0.011). In a second experiment, fecundity was compared between infected and uninfected *M. pygmaeus* females. Infection status had no effect on the amount of eggs laid (P=0.575), nor on the oocyte counts of dissected females (P=0.069).

**Table 4:** Nymphal developmental time, adult weight, sex ratio, number of eggs laid in the first week and oocyte counts of infected and uninfected *M. pygmaeus.*

Cross	Developmental time (days)		Adult weight (mg)		Sex ratio (♂ : ♀)	No. of eggs laid	Weighted sum of oocytes
		
	Males (n)	Females (n)	Males (n)	Females (n)			
I♂ x I♀	17.61 ± 0.13 a (28)	18.04 ± 0.20 a (23)	0.82 ± 0.02 a (28)	1.31 ± 0.02 a (23)	1 : 0.8	12.33 ± 1.60 a (30)	15.02 ± 0.97 a (30)
U♂ x U♀	18.54 ± 0,19 b (26)	18.60 ± 0.30 a (15)	0.83 ± 0.02 a (26)	1.19 ± 0.04 b (15)	1 : 0.6	10.96 ± 1.20 a (22)	12.44 ± 0.94 a (28)

Mean values (±SE) within a column followed by the same letter are not significantly different (P>0.05, One-Way ANOVA or Mann-Whitney U test)

## Discussion

In the present study, the microbial community of various populations of two predators of the mirid genus *Macrolophus* was investigated. The bacterial diversity of *Macrolophus* spp. was explored by cloning 16S rRNA sequences and PCR-DGGE. The cloning experiment was executed on the laboratory strain of *M. pygmaeus*, revealing the presence of bacteria from the Alpha-proteobacteria, Beta-proteobacteria, Gamma-proteobacteria and Firmicutes classes (Table 3). Three bacteria -*R. limoniae*, *R. bellii* and *Wolbachia-* can be considered as endosymbionts. The presence of these endosymbionts was confirmed using a PCR-DGGE profile of the hypervariable V3 region of the *16S rRNA* gene. The PCR-DGGE was carried out using a semi-nested approach, as the bacterial primers targeting the V3-region are known to amplify eukaryotic DNA [52]. Three bands corresponding to these three endosymbionts recurred in all studied *M. pygmaeus* populations. The DGGE-profile of bacteria in the *M. caliginosus* populations were similar to those of *M. pygmaeus*, confirming the presence of *Wolbachia* and the *Rickettsia* strain from the ‘*Limoniae’* group, but the *bellii*-like *Rickettsia* was not found (Fig. 2). A PCR using specific primers for each endosymbiont confirmed this result.

The bands with lower density present in some populations corresponded to the Gamma-proteobacteria and Firmicutes. Most of these bands were attributed to *Serratia* species of the Enterobacteriaceae family, which have been found in the gut of various insect orders, including Hymenoptera, Lepidoptera, Neuroptera and Hemiptera [53-56]. One band however (Fig. 2, no. 7), has been amplified in five wild *Macrolophus* populations. This band corresponded to an uncultured Gamma-proteobacterium, the role of which is unknown. The low bacterial diversity in the gut of *M. pygmaeus* may be attributed to its natural diet. A more diverse bacterial community is mostly detected in insects that consume nutritionally poor diets [57], whereas the main food of *Macrolophus* bugs consists of nutrient-rich arthropod prey. Also, the microbial diversity of the investigated *Macrolophus* spp. may have been underestimated by the dominance of the endosymbionts in its host. Samples of the wild *Macrolophus* populations were collected in ethanol and DNA-extraction was performed on whole adults; gut dissections were thus only feasible for the two laboratory reared populations. The faint bands in the DGGE-profile of the wild populations of *Macrolophus* may originate from prey remnants in the gut. A PCR-DGGE profile of the gut of the laboratory populations of *M. pymaeus* and *M. caliginosus* established the presence of the Gamma-proteobacteria and the *Rickettsia* endosymbionts in *M. pygmaeus* (Fig. 3), whereas the gut of *M. caliginosus* was only infected by *R*. *limoniae*. In both species, *Wolbachia* was virtually absent in the gastro-intestinal tract.

The DGGE profile of the ovaries only indicated an infection by the *Wolbachia* and *Rickettsia* endosymbionts, suggesting that no other bacteria infected the reproductive tissues. A diagnostic PCR on adults and ovaries of *M. pygmaeus* and *M. caliginosus* confirmed that all individuals are multiple infected and that the endosymbionts are vertically transmitted, implying that the infections are fixed. A FISH analysis confirmed high densities of both *Wolbachia* and *Rickettsia* in the ovarioles of *M. pygmaeus* (Fig. 4 and 5), suggesting a high rate of vertical transmission to the progeny [58].

*Wolbachia* is the only endosymbiont infecting the studied *Macrolophus* spp. which is known to cause CI in its insect host [7]. As there is no evidence in the literature that *Rickettsia* is involved in causing CI effects in insects, the strong CI observed in *M. pygmaeus *[24] is more likely related to the presence of *Wolbachia* rather than the *Rickettsia* species*.* The impact of the *Rickettsia* species on the biology of *Macrolophus* bugs is as yet unclear. A bio-assay was performed to examine differences in development and fecundity between an endosymbiont-infected and a cured population of *M. pygmaeus*. In accordance with the findings of Chiel el al. [59] on the tobacco whitefly *B. tabaci*, nymphal development of infected individuals was faster (albeit in the current study only for males), but fecundity was not affected. On the other hand, Himler et al. [60] demonstrated the rapid spread and fixation of a southwest American whitefly population infected with *Rickettsia bellii*. This population dominated all other populations by large fitness advantages and a higher proportion of females. Although the proportion of females was also higher in the infected *M. pygmaeus* population in our study (Table 4), the observed effects do not allow to explain the *Rickettsia* fixation in *Macrolophus*.. The *Rickettsia* symbiont of the booklouse *L. bostrychophila* is essential for the development of the embryos [24]. Conversely, cured *M. pygmaeus* adults produce normal progeny, confirming the facultative secondary character of *Rickettsia* in this host. Theoretically, the *Rickettsia* endosymbionts could have invaded its *Macrolophus* host by ‘hitchhiking’ with the CI-inducing *Wolbachia* endosymbiont, as CI promotes females with multiple infections [61].

Besides influencing developmental and reproductive parameters, microbial endosymbionts can affect their host in various other ways, e.g. by being nutritional mutualists. Recently, *Wolbachia* has been shown to provide a positive fitness effect in iron-restricted diets [62]. Also, the so-called ‘symbiont-mediated protection’ is an emerging topic [2,3,59]: here, insects are protected against pathogens (including viruses [51,63] and fungi [64]) or parasitoids (e.g. the braconid wasp *Aphidius* in aphids [65]) by vertically transmitted symbionts (reviewed in [3]). This protection could be a potential system for endosymbionts to preserve their infection.

To clarify the impact of the individual endosymbiont species, their hosts can be partially cured, yielding singly infected individuals. White et al. [66] used low dose antibiotics to partially cure the doubly infected parasitoid wasp *Encarsia inaron*. This wasp needed to be cured of *Wolbachia* and *Cardinium*, two endosymbionts belonging to two different classes, the Alpha-proteobacteria and Bacteroidetes respectively. However, *Rickettsia* and *Wolbachia* belong to the same family (Rickettsiaceae), which would complicate partial curing in *Macrolophus*. The role of *Wolbachia* and *Rickettsia* in *M. caliginosus* has not been demonstrated. Establishing an endosymbiont-free population and performing crossing experiments can be a first step to investigate possible reproductive effects also in the latter *Macrolophus* species.

A *Rickettsia-*specific phylogenetic tree elucidated that one *M. pygmaeus Rickettsia* endosymbiont belonged to the ‘Limoniae’ group, whereas the other is a member of the ‘Bellii’ group (Fig. 1). The *M. pygmaeus Rickettsia* endosymbiont belonging to the ‘Bellii’ group was phylogenetically closely related to the symbionts of natural prey species of the mirid predator, including the two-spotted spider mite *T. urticae*, the pea aphid *A. pisum* and the tobacco whitefly *B. tabaci*. This finding may indicate a possible horizontal transfer between predator and prey. The horizontal transfer of an endosymbiont has, however, currently only been established in an arthropod parasitoid-host system. Chiel et al. [67] investigated the interspecies horizontal transfer of *Rickettsia* from *B. tabaci* (belonging to the ‘Bellii’ group) to its aphelinid parasitoids *Eretmocerus emericus* and *E. emiratus*. This *Rickettsia* infection reached the reproductive tissues of its host, but was not transmitted to its progeny.

Sharing the same habitat and using the same plant tissues may also constitute a transmission route for bacterial endosymbionts. *Macrolophus* spp. are facultatively phytophagous predators with piercing-sucking mouthparts and may inoculate plant tissues with micro-organisms. Other species, feeding on the same host plant may then take up these micro-organisms. Furthermore, the PCR-DGGE profile showed the presence of *R. limoniae* and *R. bellii* in the gut, suggesting that an infection of the faeces is likely. However, more research is needed to confirm these hypothetical horizontal transmission routes.

## Conclusions

In this study, the microbial community of the mirid predators *M. pygmaeus* and *M. caliginosus* was explored by *16S rRNA* gene cloning and PCR-DGGE. Both species were infected with *Wolbachia* and a *Rickettsia* species related to *R. limoniae*. Furthermore, *M. pygmaeus* was infected with a *Rickettsia* species belonging to the ‘Bellii’ group. The latter is phylogenetically related to *Rickettsia* species in their arthropod prey, including *B. tabaci* and *T. urticae*, which may be indicative of a potential horizontal transmission in a predator-prey system. All endosymbionts were vertically transmitted to their progeny, as demonstrated by a FISH analysis and a diagnostic PCR on the ovaries. A bio-assay with *M. pygmaeus* indicated that infection with the endosymbionts did not have fitness costs for the predator. Further research is warranted to elucidate the role of *Rickettsia* in its *Macrolophus* host.

## Authors’ contributions

TM performed the experiments and wrote the manuscript. TM, TVL and PDC designed the experiments. TVDW and NB helped with the PCR-DGGE experiments. JAS and MN collected *Macrolophus* bugs in Spain and Italy, respectively. WDV helped with the FISH experiments. TVL, TVDW, GG and PDC revised the manuscript. All authors read and approved the final manuscript.

## Competing interests

The authors declare that they have no competing interests.

## Supplementary Material

Additional file 1 **Accession numbers phylogenetic tree.** Description: Accession numbers of the *16s rRNA*, *glta* and *coxA* genes of different species used for constructing the phylogenetic tree of *Rickettsia*. Click here for file
