# Supplementary material for: Microbial community of predatory bugs of the genus Macrolophus (Hemiptera: Miridae)
Source: BMC Microbiol. 2012 Jan 18;12(Suppl 1):S9. doi: 10.1186/1471-2180-12-S1-S9 (PMC3287520; doi:10.1186/1471-2180-12-S1-S9)
Supplement: Additional file 1 — Accession numbers phylogenetic tree. Description: Accession numbers of the 16s rRNA, glta and coxA genes of different species used for constructing the phylogenetic tree of Rickettsia. [file 1471-2180-12-S1-S9-S1.docx]

| Strain | *16s rRNA* gene | *gltA* gene | *coxA* gene |
| --- | --- | --- | --- |
| *Orientia tsutsugamushi* | D38624 | AP008981 | AM494475 |
| *Kytorhinus sharpianus* | AB021128 |  |  |
| *Tetranychus urticae* | AY753175 |  |  |
| *Bemisia tabaci* | DQ077707 | DQ077708 |  |
| *Rickettsia bellii* | CP000087 | CP000087 | CP000087 |
| *Pnigalio soemius* | EU881496 | GU559856 |  |
| *Acyrtosiphon pisum* | U42084 | FJ666756 | FJ666777 |
| *Empoasca papayae* | U76910 | U76908 |  |
| *Macrolophus* symbiont 1 | HE583203 | HE583221 | HE583222 |
| *Onychiurus sinensis* | AY712949 |  |  |
| *R. canadensis* | L36104 | CP000409 | CP000409 |
| *Coccotrypes dactyliperda* | AY961085 |  |  |
| *R. asiatica* | AF394906 | AB297810 |  |
| *R. massiliae* | L36106 | CP000683 | CP000683 |
| *R. amblyommii* | U11012 | AY375163 |  |
| *R. montanensis* | U11016 | U74756 |  |
| *R. japonica* | L36213 | U59724 |  |
| *R. honei* | NR_025967 | AF018074 |  |
| *R. peacockii* | DQ062433 | DQ100162 | CP001227 |
| *R. ricketsii* | U11021 | CP000848 | CP000848 |
| *R. conorii* | L36107 | U59730 | AE006914 |
| *R. sibirica* | D38628 | U59734 |  |
| *R. africae* | L36098 | U59733 | CP001612 |
| *R. barbariae* | EU272189 | EU272185 |  |
| *R. parkeri* | L36673 | U59732 |  |
| *R. typhi* | U12463 | U59714 |  |
| *R. prowazekii* | M21789 | U59715 | CP001584 |
| *R.tamurae* | AY049981 | AF394896 |  |
| *Ixodes scapularis* | D84558 |  |  |
| *Liposcelis bostrychophila* | DQ407744 |  |  |
| *R. felis* | CP000053 | CP000053 | CP000053 |
| *R. australis* | L36101 | U59718 |  |
| *R. akari* | L36099 | CP000847 | CP000847 |
| *Hemiclepsis marginata* | AB066352 |  |  |
| *Torix tukubana* | AB113214 |  |  |
| *Limonia chorea* | AF322442 |  |  |
| *Deronectes platynotus* | FM177877 | FM177878 |  |
| *Macrolophus* symbiont 2 | HE583202 |  | HE583223 |
